# Supplementary material for: The Increase in the Frequency and Amplitude of the Beating of Isolated Mouse Tracheal Cilia Reactivated by ATP and cAMP with Elevation in pH
Source: Int J Mol Sci. 2024 Jul 26;25(15):8138. doi: 10.3390/ijms25158138 (PMC11312401; doi:10.3390/ijms25158138)
Supplement: Supplementary file 1 [file ijms-25-08138-s001.zip › supp. captions.pdf]

Video S1: Isolated cilium without any stimulation; No beating was activated.

Video S2: Isolated cilium stimulated by 2.5 mM ATP at pH 7.4; ATP stimulation activated the repeated beating with low frequency and small amplitude.

Video S3: Isolated cilium stimulated by 2.5 mM ATP plus 10 μM 8Br-cAMP at pH 7.4; ATP plus 8Br-cAMP activated the repeated beating in an isolated cilium at pH 7.4.

Video S4: Isolated cilium stimulated by 2.5 mM ATP plus 10 μM 8Br-cAMP at pH 7.0; ATP plus 8Br-cAMP decreased the CBF and CBD at pH 7.0 compared with pH 7.4.

Video S5: Isolated cilium stimulated by 2.5 mM ATP plus 10 μM 8Br-cAMP at pH 8.0; ATP plus 8Br-cAMP increased the CBF and CBD at pH 8.0 compared with pH 7.4.

Figure S1. Electron micrograph of isolated cilia. A small number of cilia shows the 9 + 2 structure (marked by the arrows). The 9 + 2 structure was not detected in most of the cilia.
