# Supplementary figures and images for: The Increase in the Frequency and Amplitude of the Beating of Isolated Mouse Tracheal Cilia Reactivated by ATP and cAMP with Elevation in pH
Source: Int J Mol Sci. 2024 Jul 26;25(15):8138. doi: 10.3390/ijms25158138 (PMC11312401; doi:10.3390/ijms25158138)

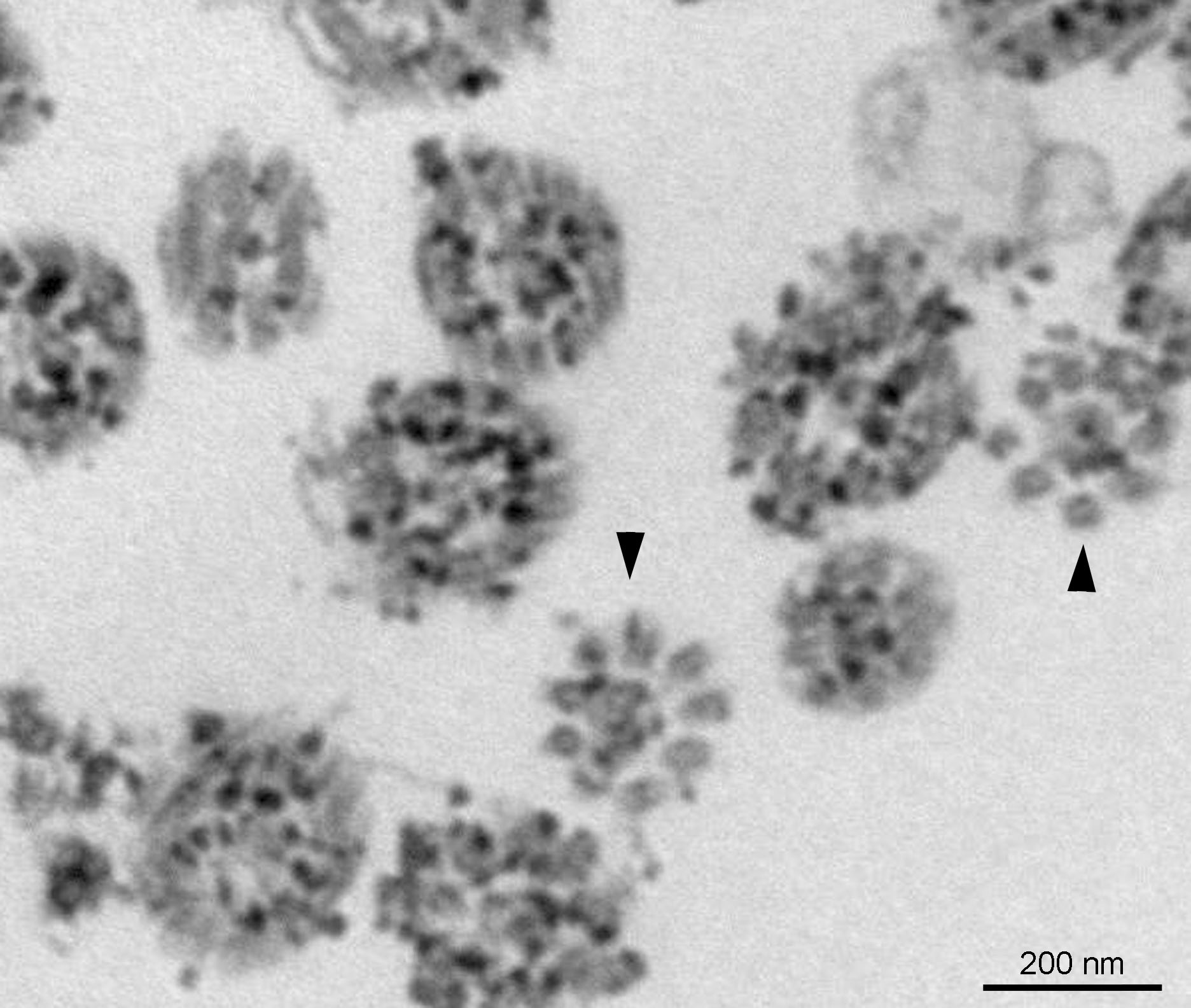

Supplement: Supplementary file 1 [file ijms-25-08138-s001.zip › Figure S1_300grey.jpg]
